# Supplementary material for: Annexin- and calcium-regulated priming of legume root cells for endosymbiotic infection
Source: Nat Commun. 2024 Dec 6;15:10639. doi: 10.1038/s41467-024-55067-3 (PMC11621553; doi:10.1038/s41467-024-55067-3)
Supplement: Supplementary file 1 — Supplementary Information [file 41467_2024_55067_MOESM1_ESM.pdf]

Annexin and calcium-regulated priming of legume root cells for endosymbiotic infection

Guillory et al.

This PDF includes:

Supplementary Figs. 1 to 16

Supplementary Table 1

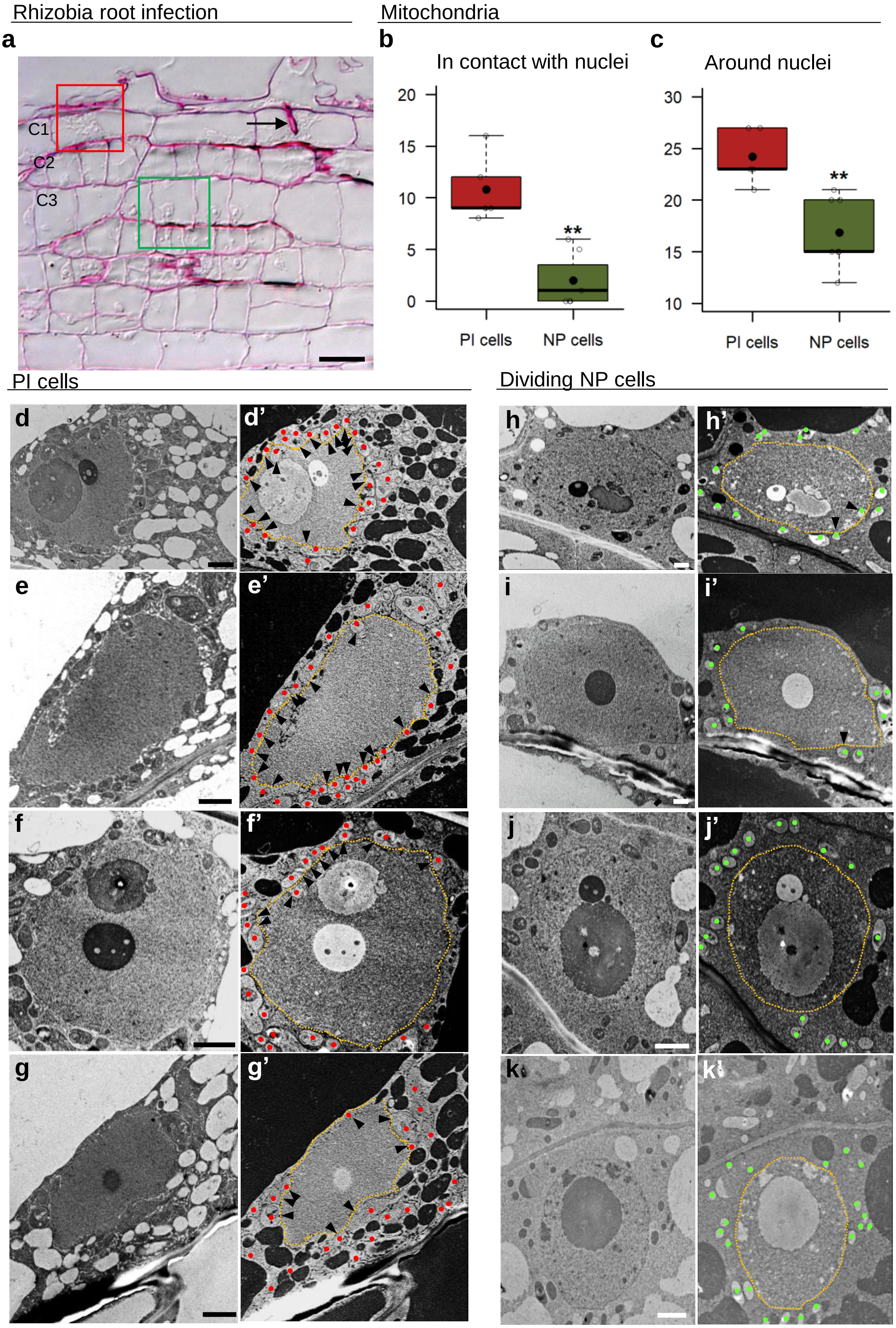

Supplementary Fig. 1. Quantification of mitochondria in the vicinity of nuclei in rhizobia pre-infection-primed (PI) and dividing nodule primordia (NP) cells (related to Fig. 1).

(**a**) A representative image of a 1 µm longitudinal section of an early rhizobia root colonization site at 5 dpi shows a cortical C1 pre-infection primed cell (red square) and another cortical C1 cell during IT penetration (arrow). (**b**-**c**) The number of mitochondria in close contact with the nuclear envelope (**b**) or within a 2 µm radius around the nucleus (**c**) were quantified in images of 80 nm-sections of C1-C2 cells in a pre-infection state (PI cells, red box plots) or in dividing C3-C5 nodule primordia cells (NP cells, green box plots).

Measurements were performed on n = 5 PI cells (8 sections analyzed) and n = 7 NP cells (7 sections analyzed) from 3 plants and 3 independent experiments. Box plots represent the distribution of individual values (open circles). First and third quartile (horizontal box edges), minimum and maximum (outer whiskers), median (centreline), mean (solid black circle) and outliers (crosses) are indicated. Asterisks in **b**-**c** indicate statistically significant differences in PI vs. NP cells ($p = 0.0054$, two-tailed Mann Whitney test in **b**, $p = 0.0026$, two-tailed Student t-test in **c**). (**d**-**k**) Representative images of PI cells (**d**-**g**) or dividing NP cells (**h**-**k**), used to quantify mitochondria in **b**-**c**. (**d'**-**k'**) LUT inverted images of **d**-**k.** Mitochondria are indicated by red dots in PI cells and by green dots in NP cells, black arrowheads indicate mitochondria in contact with the nucleus and yellow dotted lines underline nuclear contours. Scale bars: **a** = 50 µm, **d**-**k** = 2 µm. Source data of infection sites with primed and control cells are provided as a Source Data file.

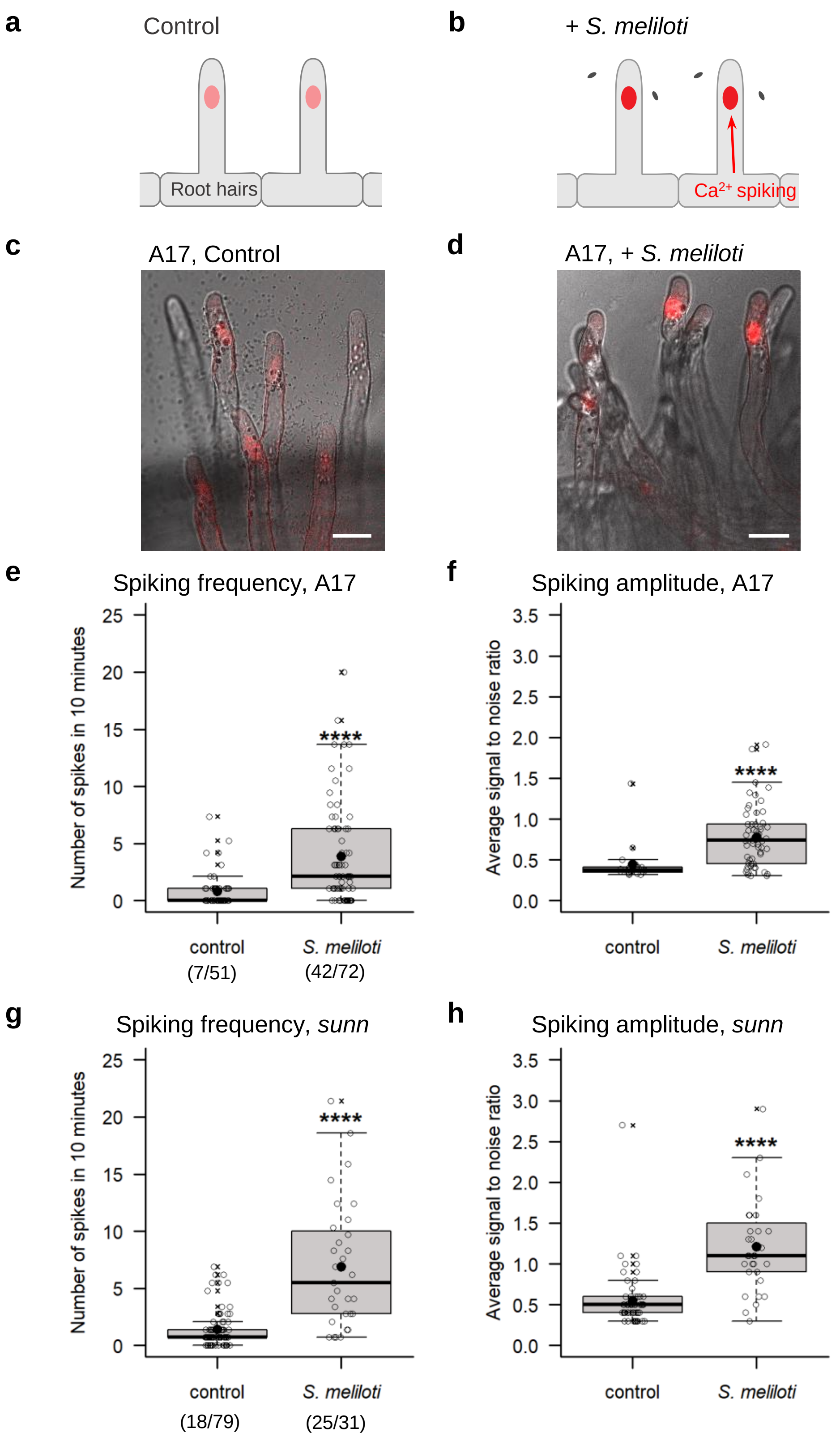

Supplementary Fig. 2. Root hair Ca^{2+} spiking responses to rhizobial inoculation in A17 and *sunn* (related to Fig. 2).

(**a**-**d**) Schematic illustration (**a**-**b**) and representative confocal images (**c**-**d**) of nuclear NR-GECO1 fluorescence (in red) in Control, non-inoculated (**a**, **c**) and *S. meliloti*-inoculated but non-infected (**b**, **d**) A17 root hairs of the nodulation-susceptible root zone 1 dpi. Images in **c**-**d** are part of Supplementary movies M1 and M2, respectively. (**e**-**h**) Spiking frequency (**e**, **g**), expressed as number of spikes in 10 minutes per nucleus and spiking amplitude (**f**, **h**), expressed as average signal-to-noise ratio (SNR, cf. Methods section) of spikes per nucleus, in A17 (**e**, **f**) and *sunn* (**g**, **h**).

Box plots in **e**, **g** represent the distribution of individual values from (**e**) Control (n = 51) and inoculated (+ *S. meliloti*, n = 72) A17 root hairs, and (**g**) Control (n = 79) and inoculated (+ *S. meliloti*, n = 31) *sunn* root hairs. Parentheses indicate number of root hairs with spiking/total number of root hairs. Root hairs are counted as spiking when showing more than 2 peaks in 10 min. Box plots in **f**, **h** represent the distribution of average values for root hairs showing at least 1 peak i.e. from (**f**) Control (n = 20) and inoculated (+ *S. meliloti*, n = 56) A17 root hairs and (**h**) Control (n = 63) and inoculated (+ *S. meliloti*, n = 31) *sunn* root hairs. Individual values (open circles) from 4 independent biological experiments for A17 (1 dpi) and 2 independent biological experiments for *sunn* (2-4 dpi) are represented in box plots in **e**-**h**. First and third quartile (horizontal box edges), minimum and maximum (outer whiskers), median (centreline), mean (solid black circle) and outliers (crosses) are indicated. Asterisks in **e**-**h** indicate statistically significant differences in control vs. *S. meliloti*-inoculated samples (in **e**, p = 1.811e-07; in **f**, p = 2.893e-05, in **g**, p = 3.377e-09, in **h**, p = 1.926e-09, two-tailed Mann-Whitney tests). Scale bars in **c**-**d** = 20 µm. Source data are provided as a Source Data file.

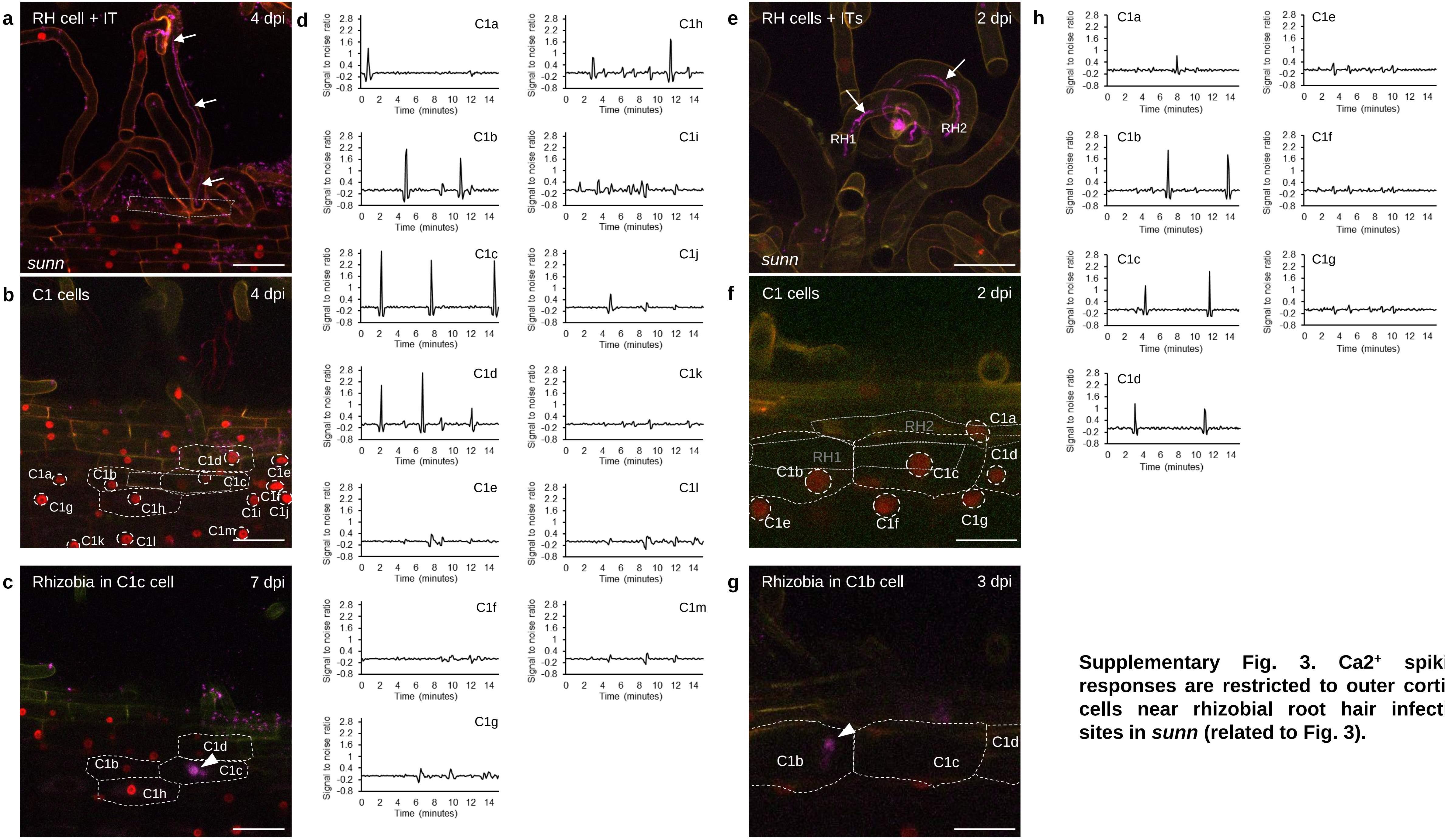

Supplementary Fig. 3. Ca^{2+} spiking responses are restricted to outer cortical cells near rhizobial root hair infection sites in *sunn* (related to Fig. 3).

Supplementary Fig. 3. Ca^{2+} spiking responses are restricted to outer cortical cells near rhizobial root hair infection sites in *sunn* (related to Fig. 3).

Ca^{2+} spiking responses were recorded in primed outer cortical cells neighbour to an infected root hair site in *M. truncatula sunn* roots expressing NR-GECO1 Ca^{2+} sensor 2-7 dpi with CFP-expressing *S. meliloti* (magenta). Representative images and Ca^{2+} spiking traces collected from two independent rhizobia infection sites are shown (**a**-**d** and **e**-**h**, respectively). (**a**, **e**) Root hair (RH) infection threads (ITs, arrows) are indicated. (**b**, **f**) Outer cortical cells located nearby infected root hairs. The positions of the infected root hair(s) epidermal bodies are indicated as grey dotted lines. Outer cortical nuclei (C1a-m in **b**, C1a-g in **f**) analysed for Ca^{2+} spiking (**d**, **h**) are indicated (round/oval dotted shapes). Non-labelled nuclei are either epidermal nuclei or those that were not in focus during the NR-GECO1 fluorescence acquisition. (**d**, **h**) Ca^{2+} spiking traces from the nuclei of the 13 (**d**) and 7 outer cortical cells (**h**) labelled in **b** and **f**. At both infection sites, only a few cortical cells situated near the epidermal bodies of the infected root hair(s) are spiking at low frequency (cells C1b-d and C1h in **b**, cells C1b-d in **f**, their contours are indicated). Among them, C1c and C1b cells in **b**-**c** and **f**-**g,** respectively, are those that host an infection thread at a later timepoint (arrowheads in **c**, **g**). Data are representative of 6 sites in 6 plants from 3 independent experiments. Ca^{2+} spiking traces in **d**, **h** are expressed as signal-to-noise ratio (SNR). Images in **a**-**c** and **e**-**g** are maximal z-projections of sub-stacks. Scale bars = 40 µm. Source data, including split channels for merged fluorescence, are provided as a Source Data file.

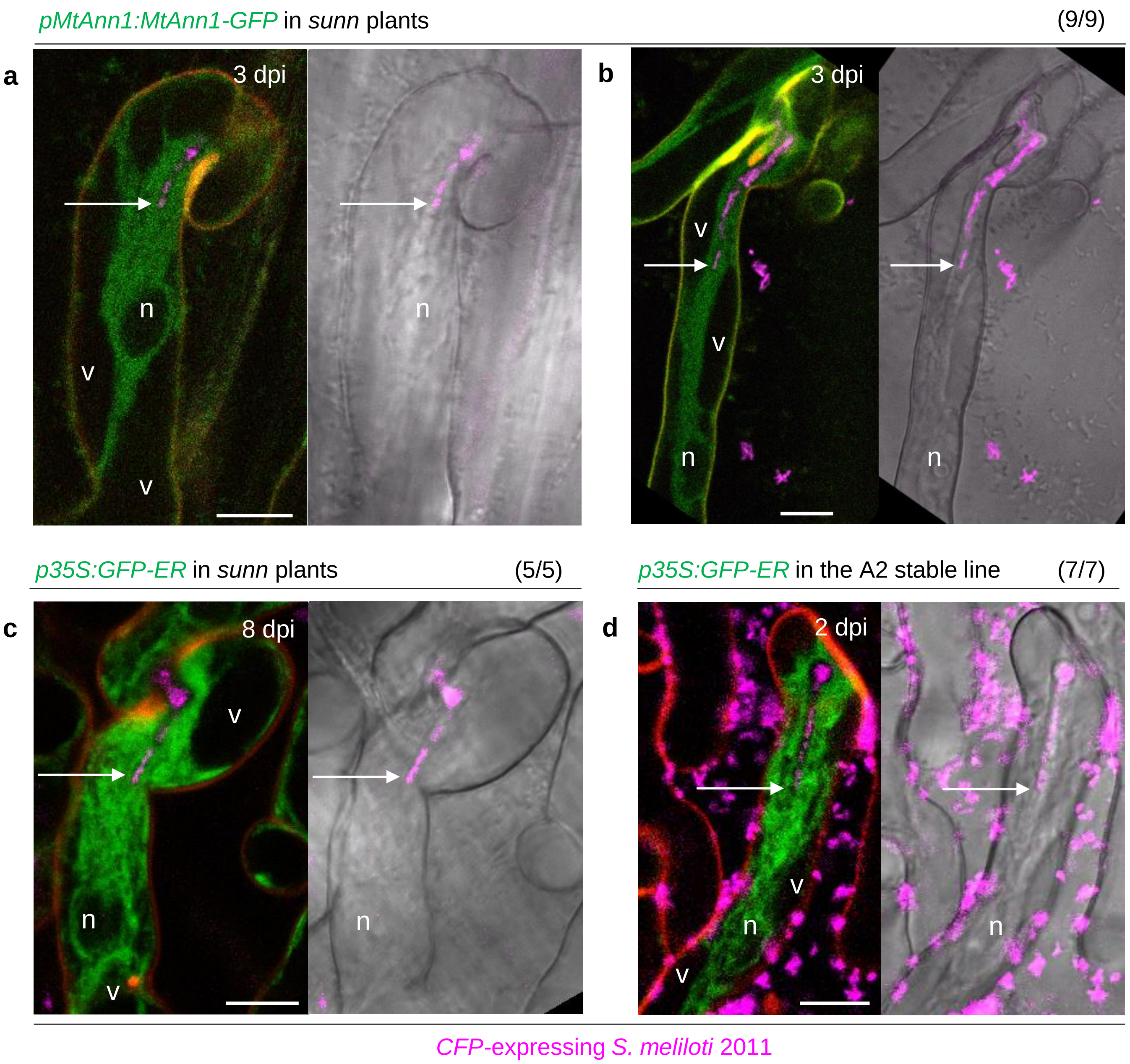

Supplementary Fig. 4. MtAnn1-GFP and GFP-ER marker label root hair cytoplasmic bridges (related to Figs. 2-3).

Root hairs undergoing infection were imaged 2 to 8 dpi with CFP-expressing *S. meliloti* (in magenta). Images were taken from root hairs of *M. truncatula sunn* composite plants expressing *pAnn1:Ann1-GFP* (**a**, **b**) or *p35S:GFP-ER* (**c**) or a stable line (referred to as A2) expressing *p35S:GFP-ER* (**d**). In these root hairs, elongating ITs are immersed in a cytoplasmic bridge connecting the IT to the nucleus (n). The cytoplasm bridges delimited by the vacuole (v, in black in the fluorescence images) are entirely and similarly labelled by MtAnn1-GFP (**a**, **b**) or GFP-ER (**c**, **d**). Arrows indicate the tip of the bacteria file within the growing infection thread. Images are merges of GFP (green), CFP (magenta) and autofluorescence (red) in left panels, or merged CFP and bright field in right panels. Parentheses (**a**-**d**) indicate the number of sites showing this pattern/total number of documented sites from 4 (**a**-**b**), 1 (**c**) and 3 (**d**) independent experiments, respectively. All sites were observed again later to confirm elongation of the imaged ITs. Scale bars = 10 µm. Source data, including split channels for merged fluorescence, are provided as a Source Data file.

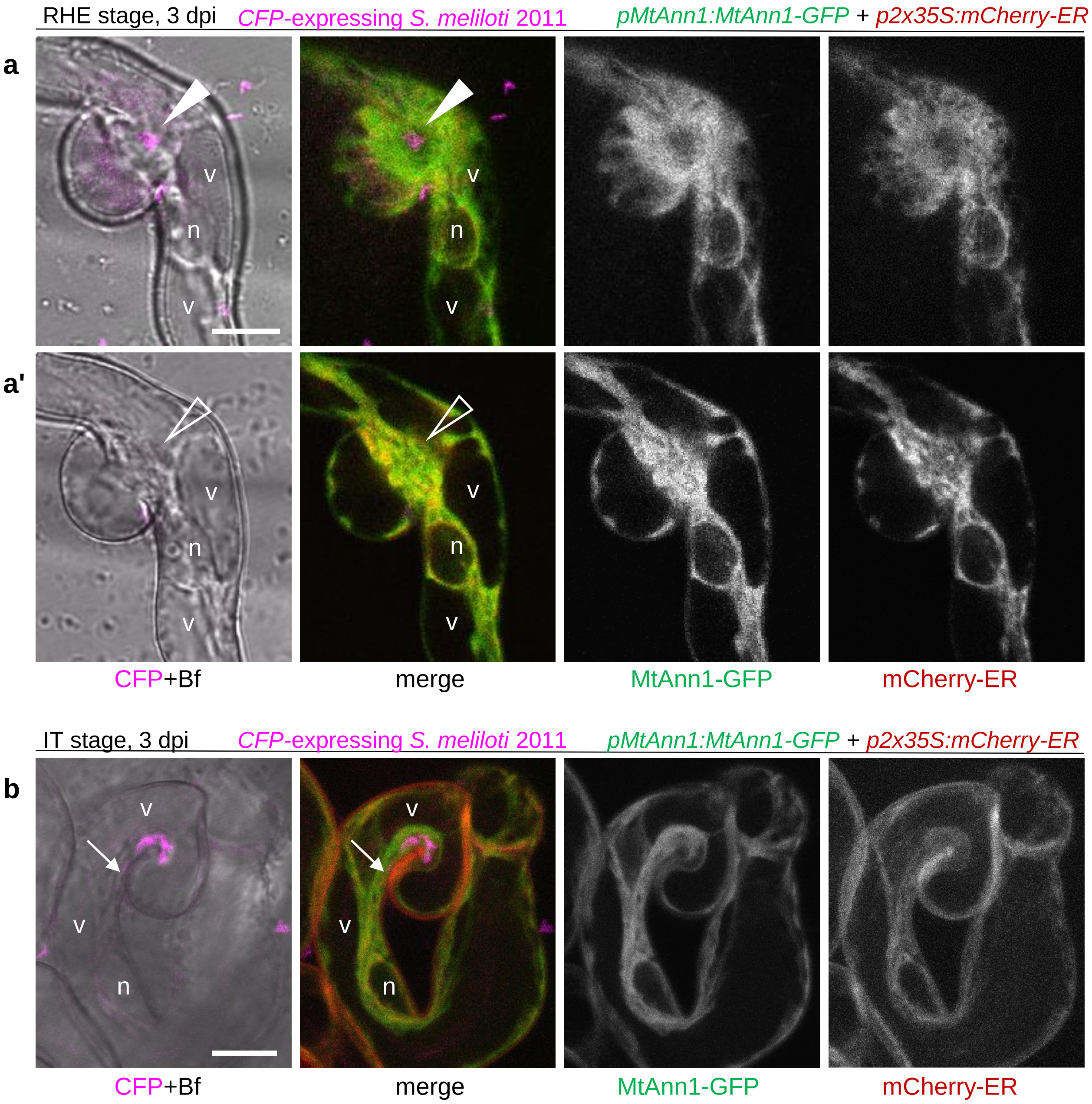

Supplementary Fig. 5. MtAnn1-GFP and mCherry-ER both label cytoplasmic bridges in *M. truncatula* root hairs (related to Figs. 2-3).

Composite *sunn* plants expressing both *pAnn1:Ann1-GFP* and *p2x35S:mCherry-ER* were inoculated with CFP-expressing *S. meliloti* (in magenta). Root hairs at early stages of infection (RHE and IT) were imaged at 3 dpi. Merged bright-field (Bf) and CFP (in magenta) are shown in the first column. Ann1-GFP and mCherry-ER fluorescences (in green and red, respectively) are merged in the second column, and showed separately (in grey) in the third and fourth columns as indicated. Colocalization of both markers in the second column is visualized in yellow. (**a**, **a’**) RHE stage, a curled root hair branch displaying an active, rhizobia-colonized infection chamber (**a**, arrowhead). **a’** shows a distinct focal plane of the same root hair as **a**, focused on the cytoplasmic bridge linking the infection chamber and the nucleus. This cytoplasmic bridge is highlighted similarly by MtAnn1-GFP and mCherry-ER. (**b**) IT stage, a curled RH hosting a developing IT (arrow). The cytoplasm in this RH, including the column linking the developing IT and the nucleus, is marked by MtAnn1-GFP and mCherry-ER alike. The site in **a**, **a’** is representative of 3 such sites in 2 independent experiments. **b** confirms the MtAnn1-GFP and GFP-ER patterns observed independently in other experiments. Images in **a** are from a single section, images in **a’** and **b** are maximal z-projections of 2 and 3 successive sections, respectively. Filled arrowhead, rhizobia in the infection chamber, open arrowhead, relative position of the infection chamber, arrow, tip of the rhizobia file within the infection thread, n, nucleus, v, vacuole. Scale bars = 10 µm. Source data are provided as a Source Data file.

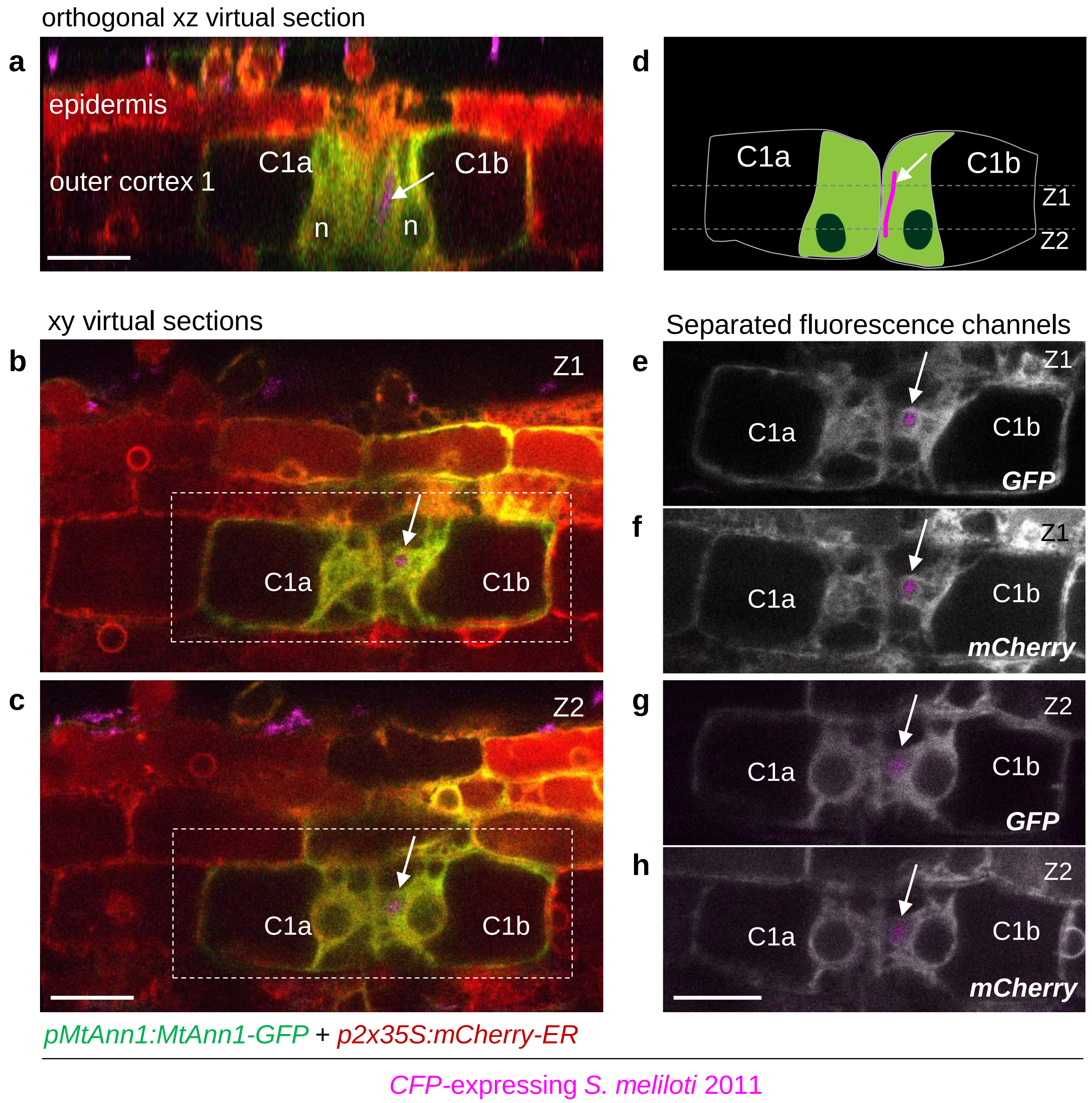

Supplementary Fig. 6. ER marker and MtAnn1-GFP co-label cytoplasmic bridges in pre-infection primed cortical cells (related to Figs. 2-3).

Composite *sunn* plants co-expressing *pAnn1:Ann1-GFP* and *p2x35S:mCherry-ER* were inoculated with CFP-expressing *S. meliloti* (in magenta). Images in the left column (**a**-**c**) are merges of CFP (magenta), GFP (green), and mCherry (red), while images in the right column are merges of CFP (magenta) and either GFP (**e**, **g**) or mCherry (**f**, **h**) in grey. (**a**) Side view (or orthogonal xz view) of two outer cortical cells, C1a and C1b. These cells are adjacent to an infected root hair (not shown) and the C1a cell is preparing for infection while C1b is being infected as revealed by the presence of a segment of the infection thread (arrow). (**b**, **c**) show two distinct optical xy sections (Z1 and Z2) across the C1a and C1b cells, as indicated in the cartoon (**d**). (**e**-**h**) show separate GFP and mCherry channels for section Z1 (**e**, **f**) and Z2 (**g**, **h**), respectively. The typical cytoplasm organization in the activated C1a cell and infected C1b cell is similarly labelled by MtAnn1-GFP (**e**, **g**) and mCherry-ER (**f**, **h**). All images in **b**-**c** and **e**-**h** are single optical sections. Arrow, rhizobia in the infection thread, n, nucleus. Scale bars = 20 µm. These images are representative of 3 primed cell sites and 6 infected outer cortex cell sites acquired in 5 plants in 1 experiment. Source data, including split channels for merged fluorescence, are provided as a Source Data file.

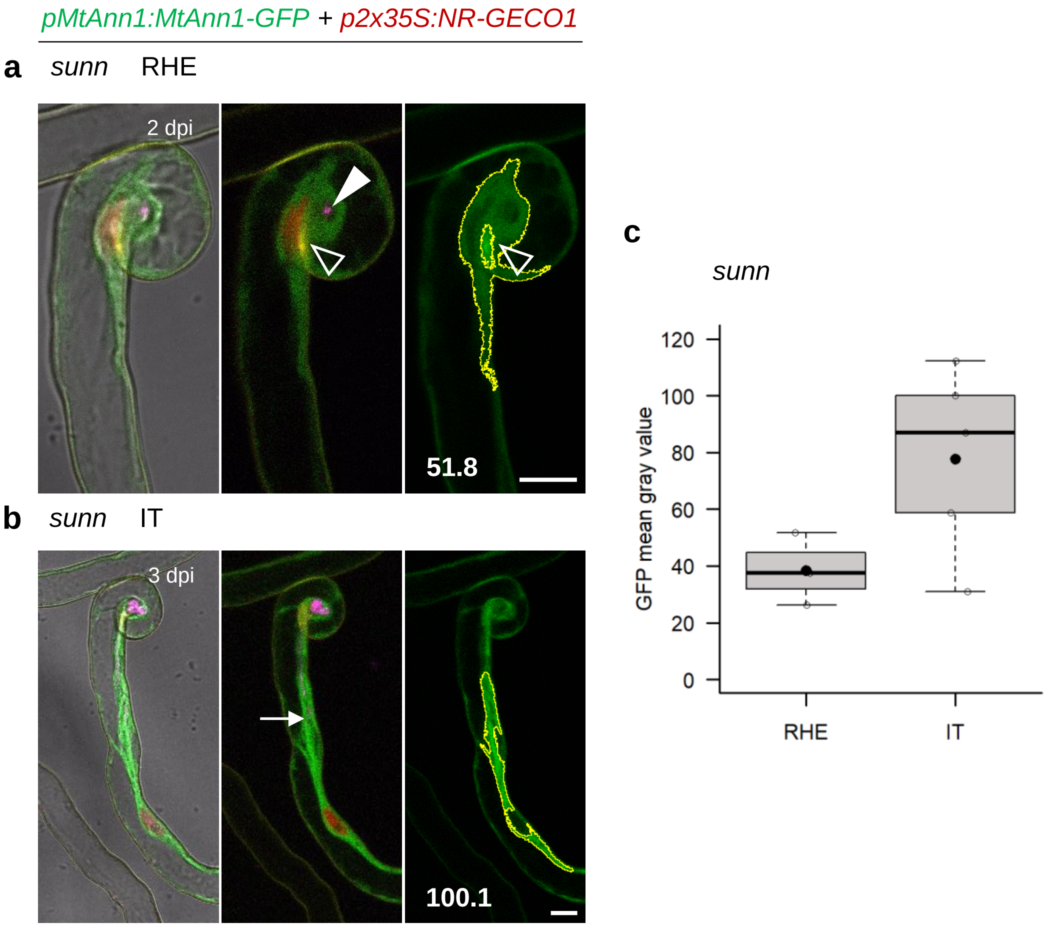

Supplementary Fig. 7. MtAnn1-GFP signal intensity in RHE and IT root hair cells (related to Fig. 2).

(**a**, **b**) MtAnn1-GFP fluorescence intensity was measured in root hairs at RHE (root hair with entrapped rhizobia) or IT (root hair with a growing infection thread) stages in *M. truncatula sunn*, representative images are shown in **a** (RHE) and **b** (**IT**). Root hairs were imaged in roots expressing MtAnn1-GFP (green) and NR-GECO1 (red), after inoculation with CFP-expressing *S. meliloti* (magenta). Merges of CFP, GFP and NR-GECO1 with (left panels) or without (central panels) bright field images are shown. Right panels show GFP fluorescence alone and the contours (yellow lines) of the areas (ROIs) used to measure mean grey fluorescence levels (numbers in lower left corner) in the cytoplasm aggregated around the site of rhizobia entrapment (**a**, arrowhead) or the cytoplasmic bridge linking an elongating infection thread (**b**, arrow) and the nucleus. Note that in this site, a second, smaller ROI (open arrowhead), corresponding to the auto-fluorescent wall domain (or contact point) adjacent to infection chambers in curled root hairs[26] was subtracted from the analysis. Arrowhead, site of rhizobia entrapment, open arrowhead, strongly auto-fluorescent wall contact point, arrow, infection thread. Scale bars = 10 µm. (**c**) Box plot represent the distribution of individual values (open circles) from RHE (n = 3) and IT (n = 5) *sunn* root hairs from 3 experiments. First and third quartile (horizontal box edges), minimum and maximum (outer whiskers), median (centreline), mean (solid black circle) and outliers (crosses) are indicated. Higher fluorescence values are observed in the IT samples, but due to their low contingency, the differences are not statistically significant (p = 0.1017, two-tailed Student t-test). Source data, including split channels for merged fluorescence, are provided as a Source Data file.

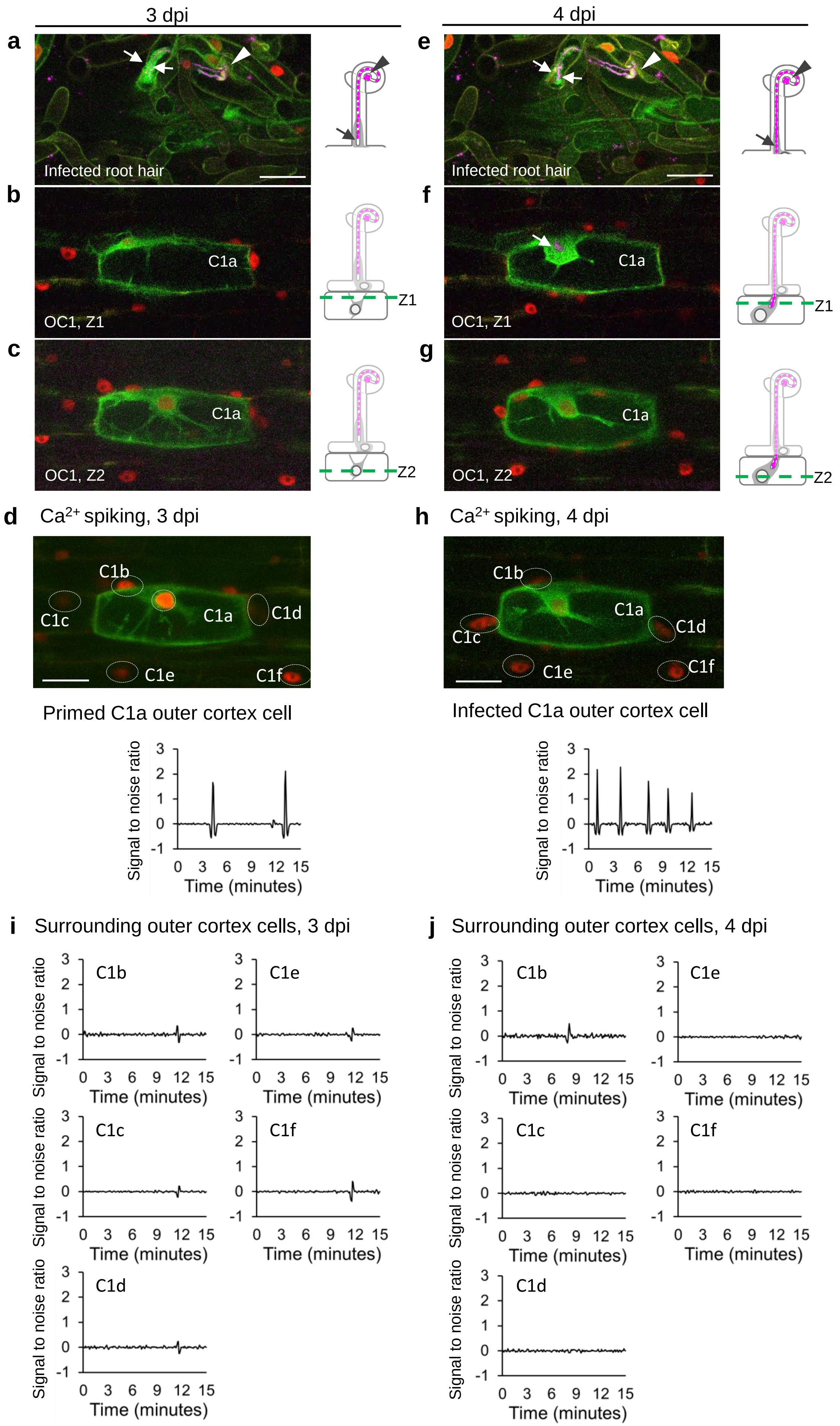

3 dpi
4 dpi
a
b
c
d
e
f
g
h
i
j
Infected root hair
OC1, Z1
OC1, Z2
C1a
Z1
Z2
Ca2+ spiking, 3 dpi
Ca2+ spiking, 4 dpi
C1a
C1b
C1c
C1d
C1e
C1f
Primed C1a outer cortex cell
Infected C1a outer cortex cell
Signal to noise ratio
Time (minutes)
Surrounding outer cortex cells, 3 dpi
Surrounding outer cortex cells, 4 dpi

Supplementary Fig. 8. MtAnn1-GFP dynamics and Ca^{2+} spiking in primed and infected cortical cells in *sunn* (related to Fig. 3).

(**a**-**j**) MtAnn1-GFP dynamics and Ca^{2+} spiking in cortical cells of *sunn* expressing *pMtAnn1:MtAnn1-GFP* (green) and *NR-GECO1* (red) below an infected root hair site (**a**-**e**) at two successive stages, 3 dpi (cortical primed state **b**-**c**) and 4 dpi (cortical infection, **f**-**g**) with CFP-labelled *S. meliloti* (magenta). **a**-**c** and **e**-**g** associate confocal images (left panels) and cartoons (right panels) of the different cell layers and features of cells preparing for infection (**b**-**c**) or infected (**f**-**g**). (**d**, **h**) illustrate the regions of interest (ROIs, dotted oval shapes) used for Ca^{2+} spiking analysis (**d**, **h**-**j**). (**b**-**d**, **f**-**h**) focus on the C1a outer cortical cell next to the infected root hair before (at 3 dpi) and during (at 4 dpi) rhizobia IT progression (**f**, arrow, Z1 section). Two virtual sections (Z1, Z2) across C1a illustrate cytoplasmic reorganisation in the pre-infection primed state (**b**-**c**) and later during infection (**f**-**g**). (**d**, **h**-**j**) show nuclear Ca^{2+} traces from C1a cell when primed to be infected (3 dpi, **d**) or during infection (4 dpi, **h**) and from surrounding outer cortical cells C1b-f (**i**, **j**). Spiking frequency is higher in C1a during IT progression (**h**) than before (primed state, **d**). At both timepoints, only the MtAnn1-GFP-labelled C1a cell is spiking. Images in **a**, **c**, **e**, **g** are maximal z-projections of 12 (**a**, **e**, **g**) or 25 (**c**) successive sections, images in **b** and **f** are single confocal sections. Images in **d** and **h** are maximal projections of whole time series. The images are representative of 3 such sites in *sunn* imaged in 3 plants from 2 experiments. Ca^{2+} traces are expressed as signal-to-noise ratio (SNR). Arrowhead, infection chamber, arrows, infection threads, RH, root hair, OC1, first outer cortical cell layer. Scale bars = 40 µm. Source data, including split channels for merged fluorescence, are provided as a Source Data file.

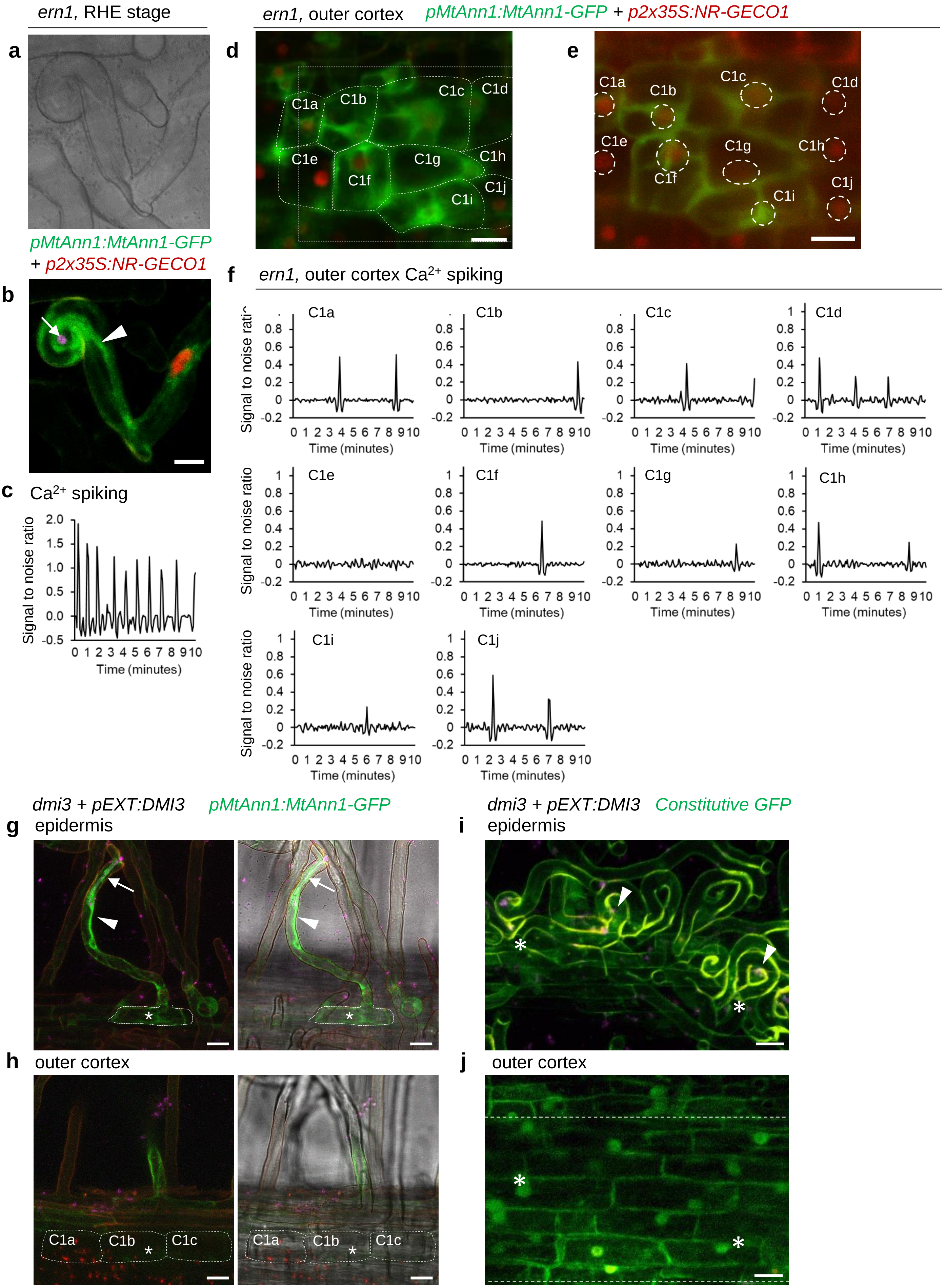

ern1, RHE stage
ern1, outer cortex pMtAnn1:MtAnn1-GFP + p2x35S:NR-GECO1
a
d
e
C1a
C1b
C1c
C1d
C1e
C1f
C1g
C1h
C1i
C1j
pMtAnn1:MtAnn1-GFP
+ p2x35S:NR-GECO1
b
f
ern1, outer cortex Ca2+ spiking
Signal to noise ratio
Time (minutes)
c
Ca2+ spiking
dmi3 + pEXT:DMI3 pMtAnn1:MtAnn1-GFP
g
epidermis
dmi3 + pEXT:DMI3 Constitutive GFP
i
epidermis
h
outer cortex
j
outer cortex

Supplementary. Fig. 9. Infection priming in symbiotic defective mutants (related to Fig. 3).

(**a**-**f**) Nuclear NR-GECO1 Ca^{2+} sensor (red) and MtAnn1-GFP fusion (green) co-expressed in *ern1* roots. (**a**-**b**) Bright-field and confocal images of an *ern1* root hair with entrapped rhizobia (RHE, 2 dpi with *S. meliloti*). Green MtAnn1-GFP fusion labels the nuclear periphery and cytoplasmic bridge (arrowhead) forming in *ern1* RHE root hairs. CFP-expressing *S. meliloti* (arrow) within the infection chamber are in magenta. (**c**) Relative intensity of NR-GECO1 fluorescence expressed as signal-to-noise ratio (SNR, cf. Methods) in the RHE hair nucleus. (**d**-**e**) Confocal images showing MtAnn1-GFP green labelling in the nuclear periphery and radiating cytoplasmic strands in outer cortical cells (C1a-c, C1f-g, C1i) near an infected *ern1* root site (4 dpi with *S. meliloti*). (**e**) Maximal z-projection of the NR-GECO1 acquisition time series (corresponding zone in **d**, grey dotted line). All outer cortical nuclei in focus are indicated (dashed white round/oval shapes). Ca^{2+} spiking traces (**f**) in *ern1* cortical cells (**d**-**e**, C1a-C1j) reflect NR-GECO1 fluorescence intensity expressed as signal-to-noise ratio (SNR, cf. Methods section). Data for *ern1* are from 3 independent experiments (n = 3). (**g**-**j**) Analysis of *dmi3* complemented with a *pEXT:DMI3* construct. (**g**-**h**) This line shows restored root hair IT development (arrow), cytoplasmic bridge formation (arrowhead) and MtAnn1-GFP expression (green signal) (**g**). Adjacent outer cortical cells C1a to C1c (**h**) show no MtAnn1-GFP labelling, unlike the wild-type A17 genotype (Fig. 3). The white asterisk indicates the position of the epidermal cell body of the infected root hair (**g**-**h**) delimited by a grey dashed line (in **g**). (**i**-**j**) Lack of cytoplasmic reorganization in the outer cortex near a rhizobia infection site (**i**, arrowheads) in the complemented *dmi3* line is also revealed by a constitutively expressed GFP marker. White asterisks (**i**-**j**) indicate the epidermal cell body position of the infected root hair. Complementation data (*dmi3* + *pEXT:DMI3*) at 6 dpi with CFP-expressing *S. meliloti* (magenta) are from 3 independent experiments (n = 11). Scale bars: **a**-**b** = 10 µm, **d**-**e**, **g**-**j** = 20 µm. Source data, including split channels for merged fluorescence, are provided as a Source Data file.

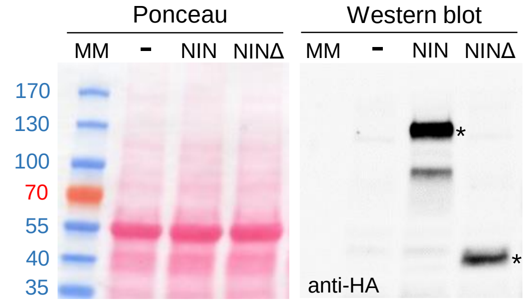

Supplementary Fig. 10. Western blot showing accumulation of 3x HA-tagged NIN versions in infiltrated *N. benthamiana* cells (related to Fig. 4).

Transactivation studies using the *pMtAnn1:GUS* fusion were performed in *N. benthamiana* leaves with or without (-) co-infiltration with 3x HA-tagged NIN (NIN) or a DNA binding domain deleted variant (NINΔ) in 4 independent experiments (see Fig. 4). Pooled protein extracts from two independent plants per sample (*pMtAnn1:GUS* -, *pMtAnn1:GUS* + NIN or *pMtAnn1:GUS* + NINΔ) were used for Western blot analysis using anti-HA antibodies (right panel). Left panel shows protein loading and the molecular marker (MM) by Ponceau staining. NIN protein bands of expected sizes are indicated by asterisks. Molecular weight sizes (kDa) are shown on the left. See Fig. 4 for corresponding transactivation experiments. Uncropped and unprocessed images for the protein gel and Western blot membrane are included in the Source Data file.

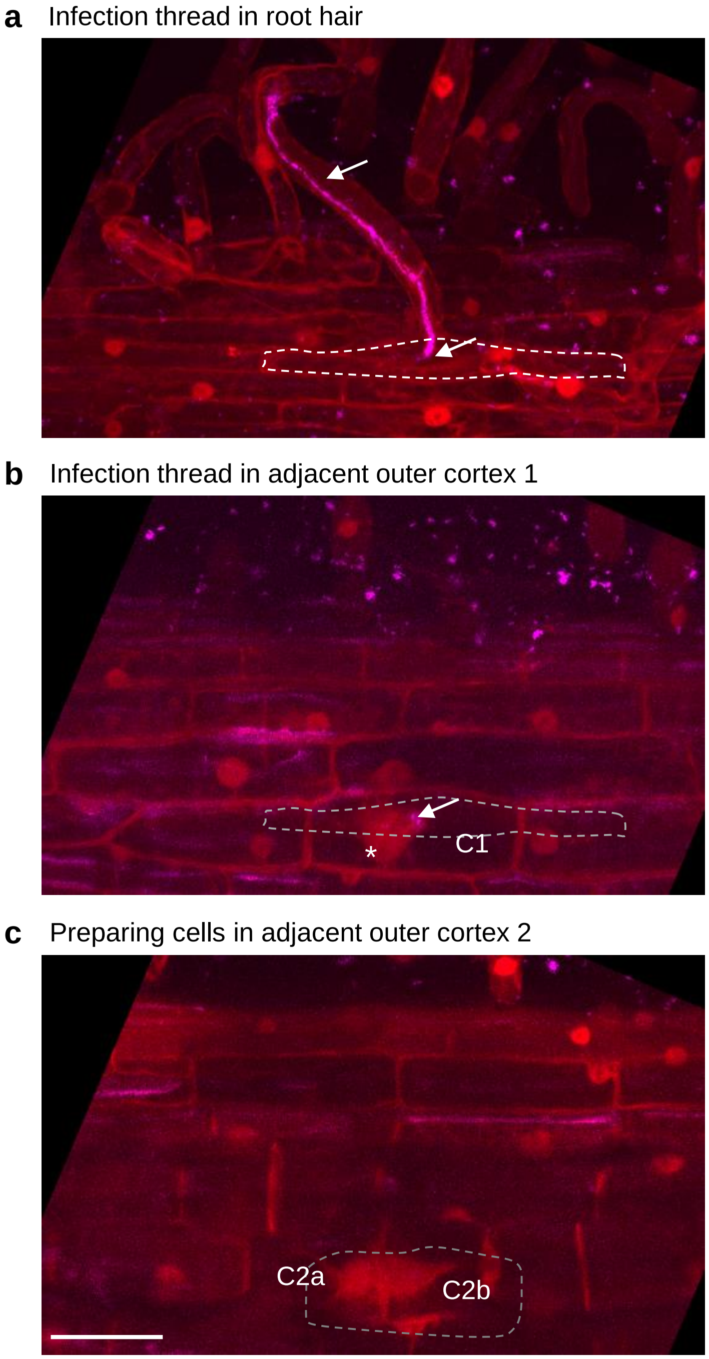

Supplementary Fig. 11. Visualization of cytoplasmic bridge formation in *sunn* expressing a *pUBQ10*-driven DsRed marker (related to Fig. 4).

Roots of *M. truncatula sunn* plants expressing *pUBQ10:DsRed* were inoculated with CFP-expressing *S. meliloti* (in magenta). The three images recapitulate the epidermal and two outer cortical cell layers (first and second, outer cortical layers 1 and 2) at a rhizobial infection site. An infection thread reaching outer cortex 1 is shown (arrows). Images are merges of DsRed (in red) and CFP (in magenta). (**a**) shows the root hair infection thread (arrows) and the position of the epidermal body of the infected root hair (dotted line). (**b**) shows adjacent outer cortex 1 cells, the C1 cell hosts the infection thread (arrow) immersed in a DsRed-labelled cytoplasmic strand close to the nucleus (asterisk). The position of the epidermal body of the infected root hair is represented by the grey dotted line. (**c**) focuses on the outer cortex 2 layer, in which two cells (C2a, C2b) directly adjacent to the infected C1 cell (depicted by the grey dotted line) display cytoplasmic rearrangement highlighted by the DsRed fluorescence. Images in **a**-**c** were obtained from a single confocal stack, as maximal z-projections of sub-stacks comprising 11 (**a**), 11 (**b**) and 7 (**c**) successive sections, respectively. Images are representative of 12 sites imaged in 7 plants in 4 independent experiments. Scale bar = 40 µm. Source data, including split channels for merged fluorescence, are provided as a Source Data file.

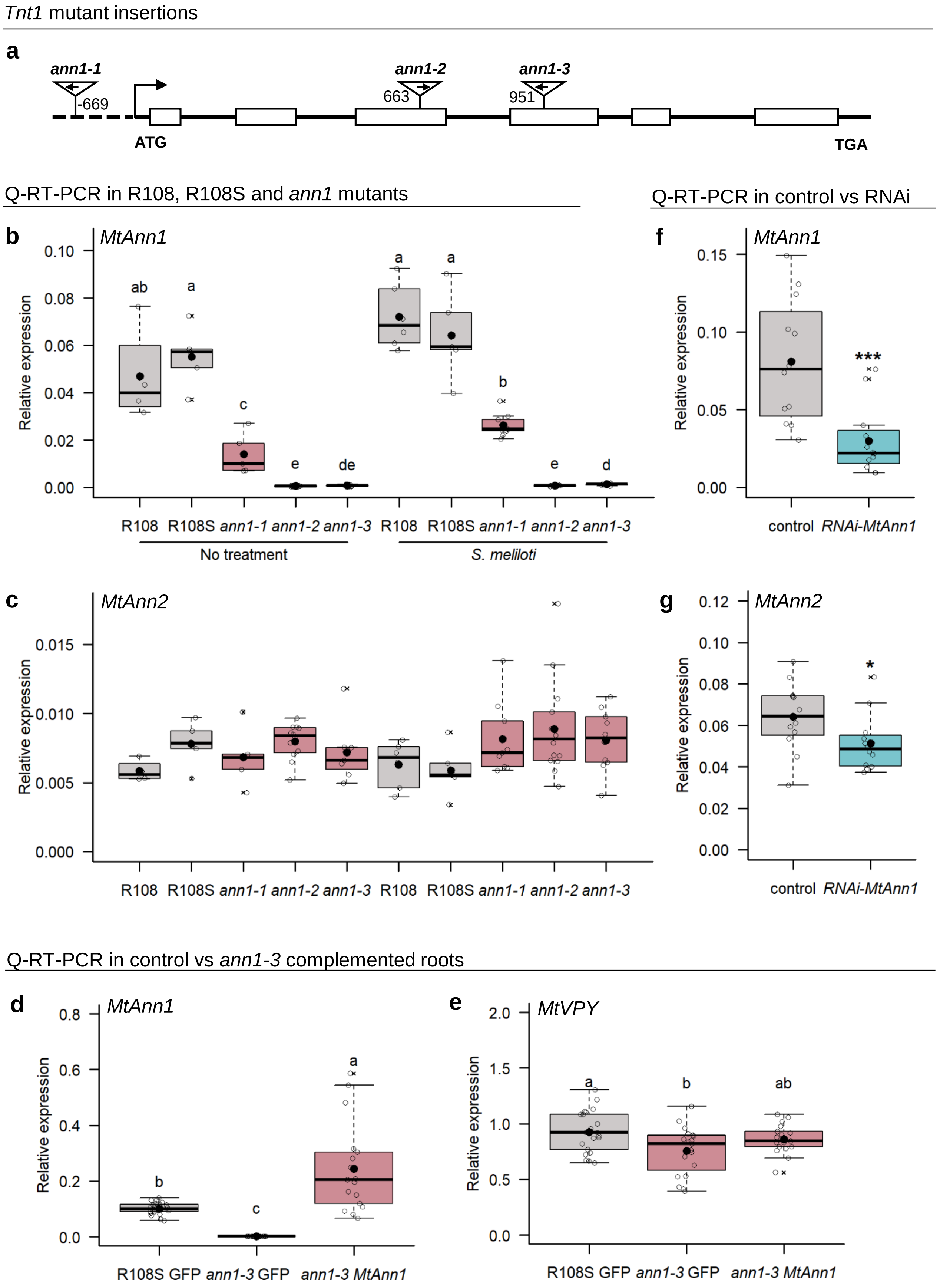
Tnt1 mutant insertions
a
ann1-1
-669
ann1-2
663
ann1-3
951
ATG
TGA
Q-RT-PCR in R108, R108S and ann1 mutants
b
MtAnn1
Relative expression
0.10
0.08
0.06
0.04
0.02
0.00
ab
a
c
e
de
a
a
b
e
d
R108 R108S ann1-1 ann1-2 ann1-3 R108 R108S ann1-1 ann1-2 ann1-3
No treatment
S. meliloti
Q-RT-PCR in control vs RNAi
f
MtAnn1
Relative expression
0.15
0.10
0.05
0.00
***
control RNAi-MtAnn1
c
MtAnn2
Relative expression
0.015
0.010
0.005
0.000
R108 R108S ann1-1 ann1-2 ann1-3 R108 R108S ann1-1 ann1-2 ann1-3
g
MtAnn2
Relative expression
0.12
0.10
0.08
0.06
0.04
0.02
0.00
*
control RNAi-MtAnn1
Q-RT-PCR in control vs ann1-3 complemented roots
d
MtAnn1
Relative expression
0.8
0.6
0.4
0.2
0.0
b
c
a
R108S GFP ann1-3 GFP ann1-3 MtAnn1
e
MtVPY
Relative expression
2.0
1.5
1.0
0.5
0.0
a
b
ab
R108S GFP ann1-3 GFP ann1-3 MtAnn1

Supplementary Fig. 12. Molecular characterization of *MtAnn1 Tnt1* insertion lines and RNAi roots (related to Figs. 5-6).

(**a**) Schematic of *ann1-1, ann1-2* and *ann1-3 Tnt1* insertion sites, -669 bp upstream of the start codon, in exon 3 or 4 in *MtAnn1*. Exons (white rectangles) and introns (black lines) are fully represented, upstream regions or *Tnt1* inserts are not. (**b**-**g**) Q-RT-PCR expression analysis of *MtAnn1* (**b**, **d**, **f**), *MtAnn2* (**c**, **g**) and *MtVPY* (**e**) in total RNA samples of non-inoculated or *S. meliloti*-inoculated roots (4 dpi in **b**-**e** and 5 dpi in **f**-**g**) from *ann1* mutants (*ann1-1*, *ann1-2* and *ann1-3*), wild-type R108 and/or R108S (**b**-**c**), R108S or *ann1-3* transformed with *p35S:GFP* (control) or *p35S:MtAnn1-GFP* (**d**-**e**) and *RNAi-MtAnn1,* control (*pMtAnn1*:GUS) plants (**f**-**g**). Transcript values were normalized against the ubiquitin reference. Data are from 2 (**b**-**e**) or 3 (**f**-**g**) independent experiments from non-treated (R108 n = 4, R108S n = 5, *ann1-1* n = 5, *ann1-2* n = 12, *ann1-3* n = 7), *S. meliloti*-inoculated samples (R108 n = 6, R108S n = 5, *ann1-1* n = 9*, ann1-2* n = 14, *ann1-3* n = 10) in **b**-**c**; R108S GFP n = 22, *ann1-3* GFP n = 19, *ann1-3 MtAnn1* n = 17 in **d**-**e**; control n = 12, *RNAi-MtAnn1* n=12 in **f**-**g**. Box plots represent distribution of individual values (open circles). First and third quartile (horizontal box edges), minimum and maximum (outer whiskers), median (centreline), mean (solid black circle) and outliers (crosses) are indicated. Values were compared in mutant vs. wild-type lines (complemented or not) in **b**-**e** and in control vs. RNAi plants in **f**-**g** by One-way ANOVA followed by Tukey HSD in **b**-**c**, **e**, Kruskal-Wallis in **d** and two-tailed Student t-tests in **f**-**g**. Classes sharing the same letter in **b**, **d**-**e** are not significantly different (p <2e-16 for **b**, p = 4.5265e-10 for **d**, p = 0.0143 for **e**). In **c**, wild-type and mutant lines were not significantly different (p = 0.106). Asterisks in **f**-**g** indicate statistically significant differences (p = 0.0002 for **f**; p = 0.0496 for **g**). Source data are provided as a Source Data file.

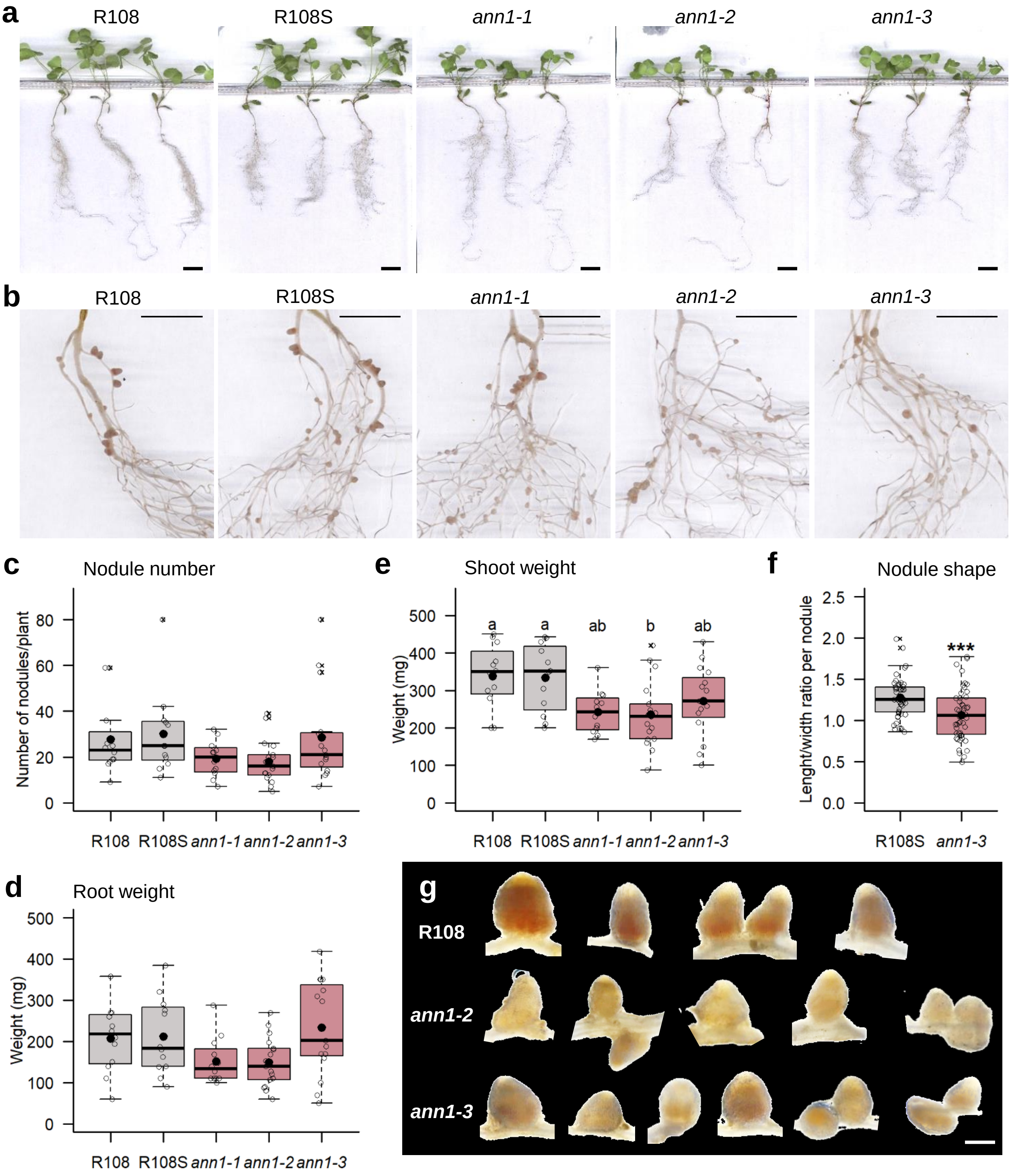

Supplementary Fig. 13. Phenotypic characterization of *MtAnn1 Tnt1* insertion lines (related to Figs. 5-6).

(**a**-**b**) Representative images of R108, R108S, *ann1-1*, *ann1-2* and *ann1-3* plants (**a**) and their nodulated root systems (**b**) at 21 dpi with *S. meliloti*. (**c**-**e**) Number of nodules per plant (**c**), fresh root weight (**d**) and fresh shoot weight (**e**) in R108 (n = 12), R108S (n = 12), *ann1-1* (n = 12), *ann1-2* (n = 17) and *ann1-3* (n = 15) at 21 dpi. (**f**-**g**) Quantification of shape (**f**) of nodules (**g**) at 21 dpi. Nodule shape (**f**), represented by the ratio of maximal length to maximal width of individual nodules, was measured on R108S (n = 43) and *ann1-3* (n = 48) mutant nodules. Box plots in **c**-**f** represent the distribution of individual values (open circles) from 3 independent experiments. First and third quartile (horizontal box edges), minimum and maximum (outer whiskers), median (centreline), mean (solid black circle) and outliers (crosses) are indicated. One-way ANOVA (**c** and **e**), Kruskal Wallis followed by a Tukey HSD test (**d**) or a two-tailed Student t-test (**f**) were performed on values. Differences between mutants and wild-type lines were not significant in **c**-**d** (p = 0.0765 in **c**, p = 0.0575 in **d**). Classes with similar letters in **e** are not significantly different (p =0.0034). Asterisks in **f** indicate statistically significant differences (p = 0.0007). Scale bars: **a** = 2 cm, **b** = 1 cm, **g** = 2.5 mm. Source data are provided as a Source Data file.

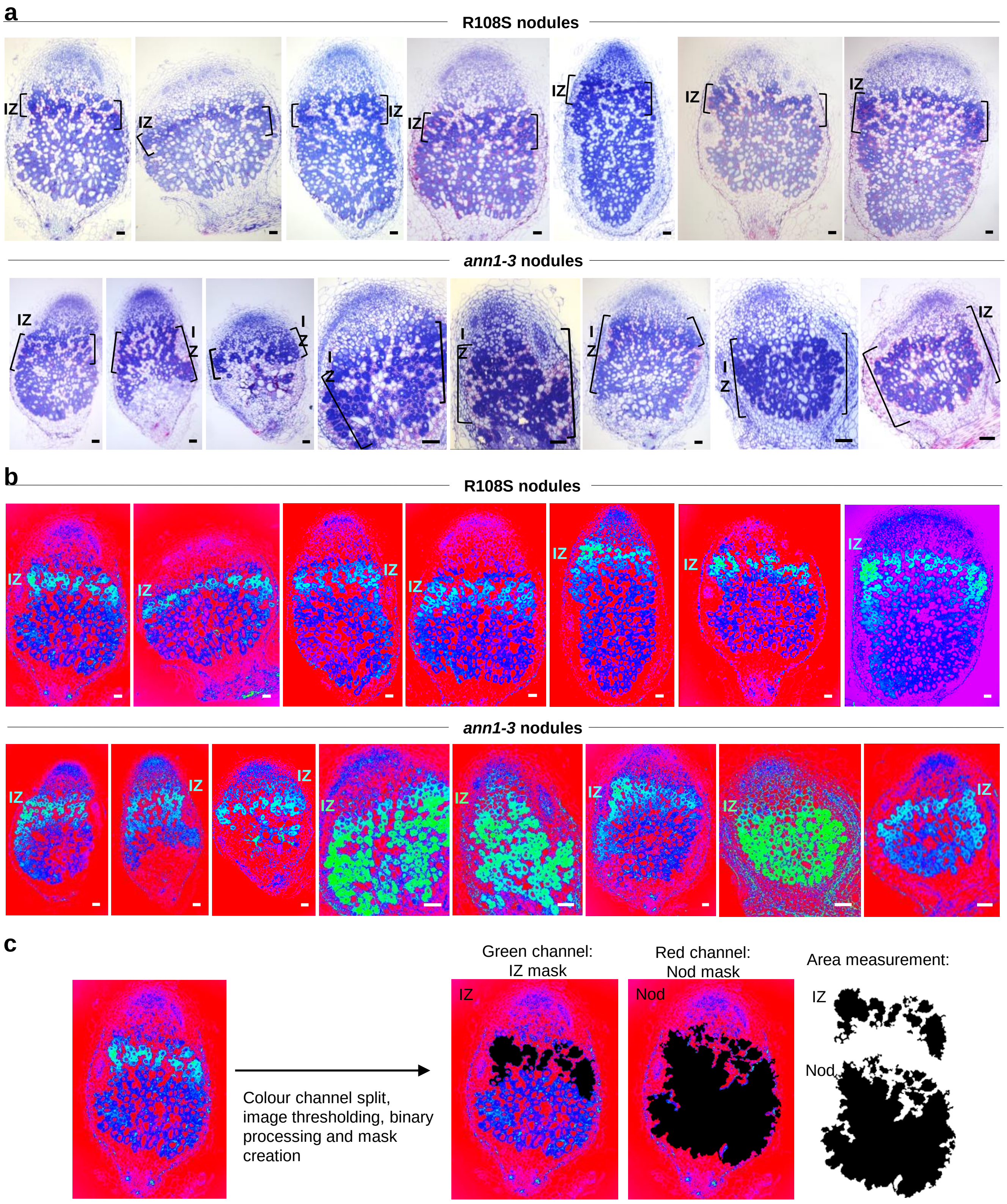

Supplementary Fig. 14. Comparative analysis of nodule zones in R108 and *ann1-3* nodules (related to Fig. 6).

(**a**-**b**) To measure the relative area of nodule interzones (IZ), bright-field images (**a**) of Toluidine Blue/Basic Fuchsin-stained 1 µm longitudinal sections of R108S (n = 7) and *ann1-3* (n = 8) nodules collected from *S. meliloti*- inoculated roots at 14-16 dpi (2 experiments) were transformed into spectrum-type image (**b**) in ImageJ, which allows better visualization of nodule IZs (in light blue to light green according to signal intensity). (**c**) Schematic representation of the ImageJ procedure used to measure the area of respective IZs and remaining nodule zones (Nod). First, spectrum images were color split and the resulting green channel (with IZ zone) and red channel (with proximal ZII, IZ and ZIII of nodules, called Nod) images were selected for further processing. Image thresholding and binary processing (fill holes) were applied to selected images to generate corresponding IZ and Nod masks (shown in black) that were then used for area measurement in ImageJ. The resulting data for IZ/Nod area per nodule is shown in Fig. 6g.

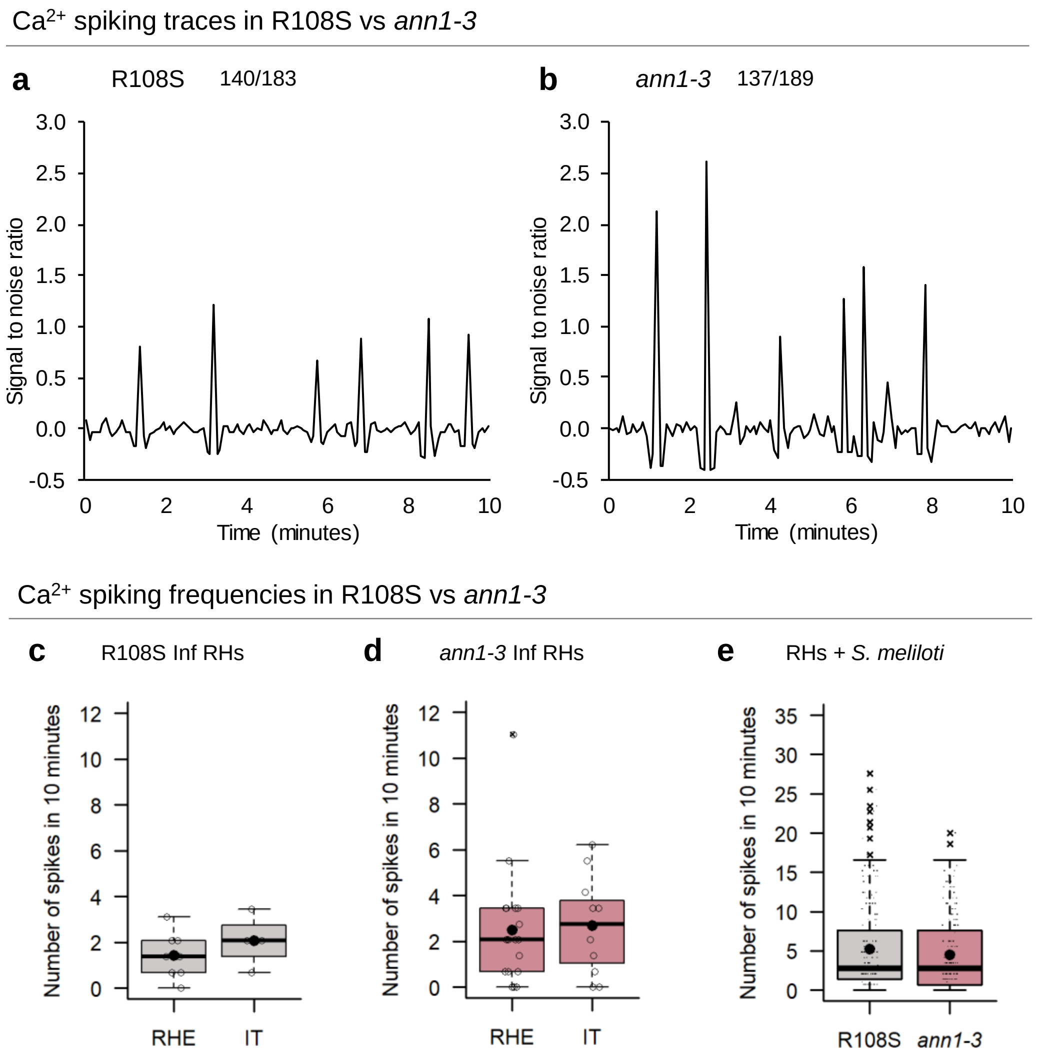

Supplementary Fig. 15. Nuclear Ca^{2+} spiking patterns in the absence of *MtAnn1* (related to Fig. 7).

Transgenic roots expressing the NR-GECO1 Ca^{2+} sensor were generated to monitor nuclear Ca^{2+} oscillations in R108S (wild-type) and in the *ann1-3* mutant. (**a**-**b**) Representative nuclear Ca^{2+} spiking traces of R108S (**a**) or *ann1-3* (**b**) in symbiotically responsive but not yet infected root hairs inoculated with *S. meliloti* at 1-4 dpi. Variations in red fluorescence of the NR-GECO1 Ca^{2+} sensor are reported as the Signal to Noise Ratio (SNR, cf. Methods section). Values on the top right correspond to the number of root hairs showing more than 2 Ca^{2+} spikes in 10 minutes, out of total root hairs analysed. (**c**-**e**) Spiking frequencies represent the number of spikes in 10 minutes for each nucleus in root hairs containing entrapped rhizobia (RHE) or ITs in R108S (RHE, n = 8; IT = 4) (**c**) or in *ann1-3* (RHE = 18; IT = 11) (**d**) backgrounds and in *S. meliloti*-responsive, non-infected root hairs (R108S, n = 183 and *ann1-3*, n = 189) (**e**). Box plots in **c**-**e** represent the distribution of individual values (open circles or black dots) from 3 independent experiments. First and third quartile (horizontal box edges), minimum and maximum (outer whiskers), median (centreline), mean (solid black circle) and outliers (crosses) are indicated. A two-tailed Student t-test (**c**) or two-tailed Mann-Whitney tests (**d** and **e**) of values did not reveal statistical differences (p = 0.3286 for **c**, p = 0.57 for **d**, p = 0.3726 for **e**). Source data are provided as a Source Data file.

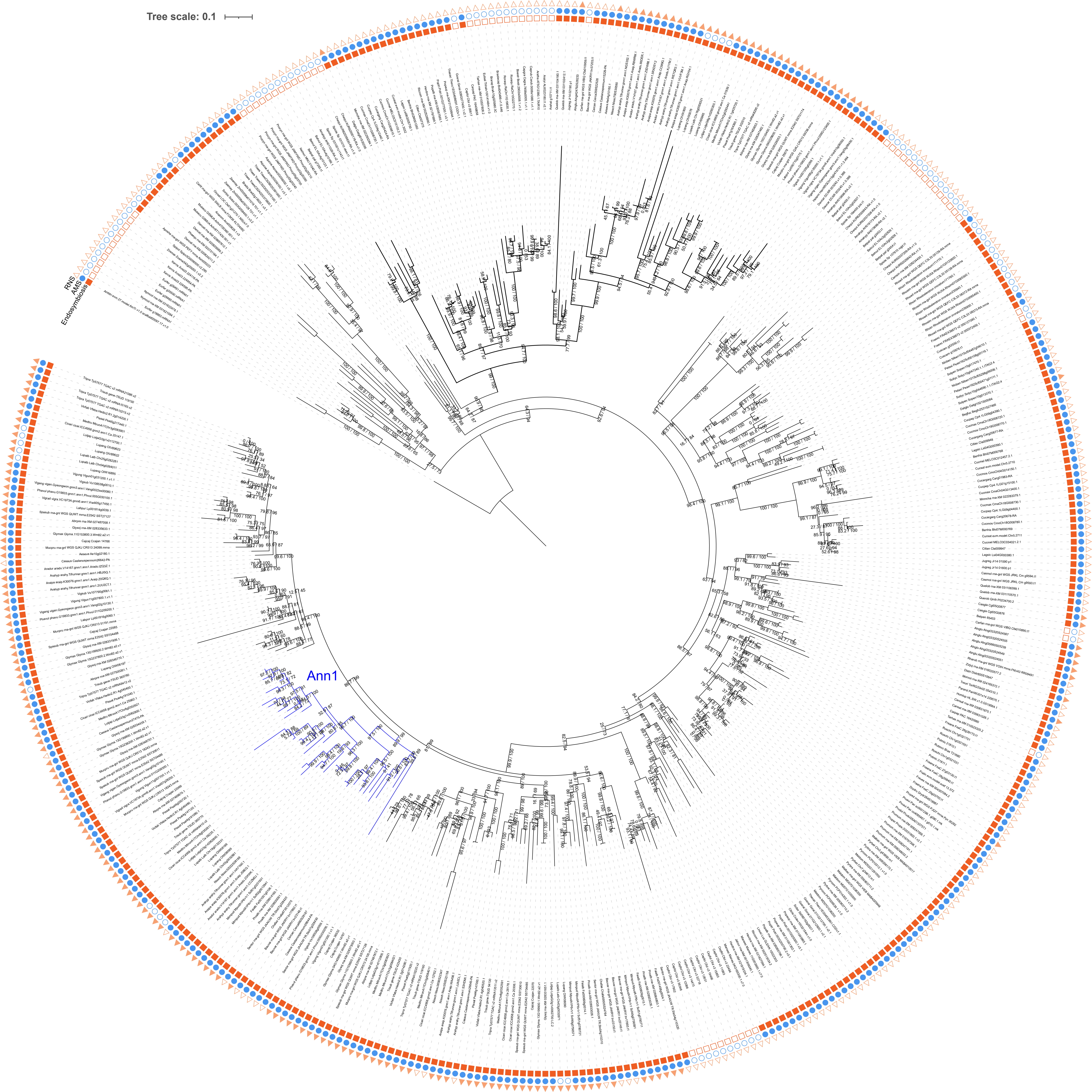
Tree scale: 0.1
RNS
AMS
Endosymbiosis
Ann1

Supplementary Fig. 16. Conservation of *MtAnn1* in root endosymbioses (related to Fig. 8).

Protein sequence of MtAnn1 (MtrunA17_Chr8g0352611) was used as query to search against a database containing 227 plant genomes covering the main lineages of green plants and five SAR (*Stramenopiles*/*Alveolata*/*Rhizaria*) genomes as outgroups[57]. Homologous proteins were then aligned using Muscle v5.1 and the phylogenetic tree was constructed using the NJ method. Plant species establishing (filled triangles, circles or squares) or not establishing (empty triangles, circles or squares) endosymbioses (orange squares) (Supplementary Data 1), AM symbiosis (blue circles) or rhizobia root nodule symbiosis (RNS) (orange triangles) are indicated.

**Supplementary Table 1. Primers used in this study.**	
***ann1* mutants genotyping primers**	
LTR4-	TACCGTATCTCGGTGCTACA
LTR6-	GCTACCAACCAAACCAAGTCAA
Mtr14183-Rev	CTCTGCTCACAATCACACGG
-344 pMtAnn1-Fw	TGATGATTTGAGTCTGGTCCCA
-914 pMtAnn1-Fw	ACCGTCCCTGTGACATTGAT
**GoldenGate cloning primers**	
pMtAnn1-GG-A-Fw	**GGTCTC**GAAATGAATTCTTTTATTGATTGCG
pMtAnn1-GG-B-Rev	**GGTCTC**GTTTGgGTTCAATATGTaTTGTTTATAg
MtAnn1-ATG-GG-B-Fw	**GGTCTC**TCAAAATGGCTACCCTTTCTGCTCCTAG
MtAnn1-STOP-GG-C-Rev	**GGTCTC**GCACCTCATTCCTCTTTCCCCAAGAGAG
MtAnn1 antisens-STOP-GG-X-Fw	**GGTCTC**GCCAGTCATTCCTCTTTCCCCAAGAGAG
MtAnn1 antisens-ATG-GG-D-Rev	**GGTCTC**GCGTAATGGCTACCCTTTCTGCTCCTAG
GUS-GG-B-Fw	**GGTCTC**TCAAAATGGTCCGTCCTGTAGAAACC
GUS-GG-D-Rev	**GGTCTC**ACGTATCATTGTTTGCCTCCCTGCTG
**qRT-PCR primers**	
1271 Ubiquitin-Fw	TTGTGTGTTGAATCCTAAGCAGG

1334 Ubiquitin-Rev	CAAGACCCATGCAACAAGTTCT
925 MtAnn2-Fw	TTGGGGAAGCAAGATTGAAG
1023 MtAnn2-Rev	CCAAACCAAACCACAAGAAAG
-5 MtAnn1-Fw	GAACTATGGCTACCCTTTCTGC
88 MtAnn1-Rev	CATCAGTACCCCATCCTTCG
MtMYB1-Fw	TAAGAGAGTTGATGATGATGTCC
MtMYB1-Rev	GATGTGTGATTCTGTTGAACC
MtVapyrin-Fw	AAACCACCATCTGCACCTTC
MtVapyrin-Rev	ACCTCTTAGCGCACGAGTTC
