# Supplementary material for: Annexin- and calcium-regulated priming of legume root cells for endosymbiotic infection
Source: Nat Commun. 2024 Dec 6;15:10639. doi: 10.1038/s41467-024-55067-3 (PMC11621553; doi:10.1038/s41467-024-55067-3)
Supplement: Supplementary file 3 — Description of Additional Supplementary Files [file 41467_2024_55067_MOESM3_ESM.pdf]

Description of Additional Supplementary files

Supplementary Data 1: List of plant species establishing or not establishing endosymbioses.

Supplementary Movie 1: GECO sensor fluorescence in control Medicago truncatula A17 root hairs.

Supplementary Movie 2: Ca2+ spiking imaged with a GECO sensor in Rhizobia-inoculated M. truncatula A17 root hairs.

Supplementary Movie 3: Ca2+ spiking visualized with a GECO sensor in dmi3 complemented in the root epidermis.
