# Supplementary material for: Annexin- and calcium-regulated priming of legume root cells for endosymbiotic infection
Source: Nat Commun. 2024 Dec 6;15:10639. doi: 10.1038/s41467-024-55067-3 (PMC11621553; doi:10.1038/s41467-024-55067-3)
Supplement: Supplementary file 8 — Reporting Summary [file 41467_2024_55067_MOESM8_ESM.pdf]

nature portfolio

Corresponding author(s): Fernanda de Carvalho-Niebel

Last updated by author(s): Nov 13, 2024

Reporting Summary

Nature Portfolio wishes to improve the reproducibility of the work that we publish. This form provides structure for consistency and transparency in reporting. For further information on Nature Portfolio policies, see our Editorial Policies and the Editorial Policy Checklist.

Statistics

For all statistical analyses, confirm that the following items are present in the figure legend, table legend, main text, or Methods section.

n/a	Confirmed	
☐	☒	The exact sample size (*n*) for each experimental group/condition, given as a discrete number and unit of measurement
☐	☒	A statement on whether measurements were taken from distinct samples or whether the same sample was measured repeatedly
☐	☒	The statistical test(s) used AND whether they are one- or two-sided<br>*Only common tests should be described solely by name; describe more complex techniques in the Methods section.*
☒	☐	A description of all covariates tested
☐	☒	A description of any assumptions or corrections, such as tests of normality and adjustment for multiple comparisons
☐	☒	A full description of the statistical parameters including central tendency (e.g. means) or other basic estimates (e.g. regression coefficient) AND variation (e.g. standard deviation) or associated estimates of uncertainty (e.g. confidence intervals)
☐	☒	For null hypothesis testing, the test statistic (e.g. *F*, *t*, *r*) with confidence intervals, effect sizes, degrees of freedom and *P* value noted<br>*Give P values as exact values whenever suitable.*
☒	☐	For Bayesian analysis, information on the choice of priors and Markov chain Monte Carlo settings
☒	☐	For hierarchical and complex designs, identification of the appropriate level for tests and full reporting of outcomes
☒	☐	Estimates of effect sizes (e.g. Cohen's *d*, Pearson's *r*), indicating how they were calculated

Our web collection on statistics for biologists contains articles on many of the points above.

Software and code

Policy information about availability of computer code

Data collection	Data were collected exclusively using commercial softwares (Leica Confocal Imaging Software, Leica Application Suite X)
Data analysis	R software (http://r-project.org), ImageJ (https://imagej.net/ij/), Ilastik (https://www.ilastik.org/), GraphPad PRISM 10.3.1

For manuscripts utilizing custom algorithms or software that are central to the research but not yet described in published literature, software must be made available to editors and reviewers. We strongly encourage code deposition in a community repository (e.g. GitHub). See the Nature Portfolio guidelines for submitting code & software for further information.

Data

Policy information about availability of data

All manuscripts must include a data availability statement. This statement should provide the following information, where applicable:

- Accession codes, unique identifiers, or web links for publicly available datasets
- A description of any restrictions on data availability
- For clinical datasets or third party data, please ensure that the statement adheres to our policy

The authors declare that all data supporting the results of this study are available in the main manuscript, Figures, Supplementary Files and Source Data file. Materials generated in this study are available from the corresponding author upon request.

Research involving human participants, their data, or biological material

Policy information about studies with human participants or human data. See also policy information about sex, gender (identity/presentation), and sexual orientation and race, ethnicity and racism.

Reporting on sex and gender	*Use the terms sex (biological attribute) and gender (shaped by social and cultural circumstances) carefully in order to avoid confusing both terms. Indicate if findings apply to only one sex or gender; describe whether sex and gender were considered in study design; whether sex and/or gender was determined based on self-reporting or assigned and methods used. Provide in the source data disaggregated sex and gender data, where this information has been collected, and if consent has been obtained for sharing of individual-level data; provide overall numbers in this Reporting Summary. Please state if this information has not been collected. Report sex- and gender-based analyses where performed, justify reasons for lack of sex- and gender-based analysis.*
Reporting on race, ethnicity, or other socially relevant groupings	*Please specify the socially constructed or socially relevant categorization variable(s) used in your manuscript and explain why they were used. Please note that such variables should not be used as proxies for other socially constructed/relevant variables (for example, race or ethnicity should not be used as a proxy for socioeconomic status). Provide clear definitions of the relevant terms used, how they were provided (by the participants/respondents, the researchers, or third parties), and the method(s) used to classify people into the different categories (e.g. self-report, census or administrative data, social media data, etc.) Please provide details about how you controlled for confounding variables in your analyses.*
Population characteristics	*Describe the covariate-relevant population characteristics of the human research participants (e.g. age, genotypic information, past and current diagnosis and treatment categories). If you filled out the behavioural & social sciences study design questions and have nothing to add here, write "See above."*
Recruitment	*Describe how participants were recruited. Outline any potential self-selection bias or other biases that may be present and how these are likely to impact results.*
Ethics oversight	*Identify the organization(s) that approved the study protocol.*

Note that full information on the approval of the study protocol must also be provided in the manuscript.

Field-specific reporting

Please select the one below that is the best fit for your research. If you are not sure, read the appropriate sections before making your selection.

☒ Life sciences ☐ Behavioural & social sciences ☐ Ecological, evolutionary & environmental sciences

For a reference copy of the document with all sections, see nature.com/documents/nr-reporting-summary-flat.pdf

Life sciences study design

All studies must disclose on these points even when the disclosure is negative.

Sample size	Individual samples were randomly collected from independent biological experiments (two to four, depending on the type of analysis, as indicated). Sample sizes were chosen on the basis of previous repeated experiments, and these sample sizes were sufficient to perform the appropriate statistical tests. Briefly, data were first analyzed for normality (using the Shapiro-Wilks test) and homogeneity of variance was assessed (using the Fisher or Bartlett tests) in order to select the appropriate statistical test (using R software). Parametric statistical tests (t-test, ANOVA) were used to analyze data with a normal distribution, while the non-parametric Mann-Whitney test was used for data with a non-normal distribution. Where necessary, a transformation was performed to normalize the data distribution (Log10 or BoxCox). The number of samples analyzed individually (n), number of independent experiments, statistical tests and significance levels p are indicated in the Figure legends and detailed in the Methods section and in the Source Data file.<br>In our experimental setups, we ensured that phenotyping of nodulation and rhizobia infection was performed after analysis of a high number of individual samples from 2-4 independent experiments. For quantification of calcium spiking responses in individual infected root hairs, we ensured that data were collected from at least 3 independent experiments. The use of the sunn genetic background (more infection sites) greatly facilitated the collection of a reasonable number of individual infected root hair samples (n=≥18) for robust statistical analysis. In addition, sites were analysed several times in vivo to validate responses over time. Calcium responses in infected R108 root hairs were obtained from a smaller number of samples (as these are technically very difficult experiments), but the data follow a normal distribution and homogeneity of variance and were obtained from 3 independent experiments (a one-tailed Student t-test was thus used in this case). However, the differences in calcium amplitude in rhizobia-inoculated root hairs of wild-type R108S compared to the annexin mutant were based on the analysis of a large number of individual rhizobia-inoculated root hairs (n=≥ 156) and were supported by robust statistical data.
Data exclusions	No data were excluded from the analyses
Replication	The experimental findings were reproducible. We had problems with biological replication in complementation experiments due to growth chamber defects. We have since then performed a new experiment and results from 2 experiments are shown (Supplementary Fig. 12).
Randomization	Medicago truncatula plants grown in different conditions were mock (control) or rhizobial-inoculated and randomly collected for further processing or analyses. For transgenic composite plants expressing different constructs, transformed roots were first selected on the basis of

antibiotic resistance and/or the expression of the fluorescent DsRed protein before mock or bacterial inoculation. Group of mock or rhizobial inoculated plants were randomly analyzed.

Blinding	Quantification of nodule size,nodule number and infection levels were also performed on scanned images of β-galactosidase-stained root systems to enable independent machine-learning mediated ImageJ quantification.

Reporting for specific materials, systems and methods

We require information from authors about some types of materials, experimental systems and methods used in many studies. Here, indicate whether each material, system or method listed is relevant to your study. If you are not sure if a list item applies to your research, read the appropriate section before selecting a response.

Materials & experimental systems

n/a	Involved in the study	
☐	☒	Antibodies
☒	☐	Eukaryotic cell lines
☒	☐	Palaeontology and archaeology
☒	☐	Animals and other organisms
☒	☐	Clinical data
☒	☐	Dual use research of concern
☐	☒	Plants

Methods

n/a	Involved in the study	
☒	☐	ChIP-seq
☒	☐	Flow cytometry
☒	☐	MRI-based neuroimaging

Antibodies

Antibodies used	anti-HA-peroxidase antibodies (Sigma-Aldrich)
Validation	Data is shown in Supplementary Fig. 10 of the manuscript. This is a comercially available antibody: https://www.sigmaaldrich.com/FR/fr/product/sigma/p7899?utm_source=google&utm_medium=cpc&utm_campaign=21473890494&utm_content=168623535247&gclid=Cj0KCQjwwae1BhC_ARIsAK4JfrwZ-C_bq1aPMT1GXST0Ng7gQLPK76WioE9Poa6EZHtA0_tS6HsngnEaAi7dEALw_wcB&icid=sharepdp-clipboard-copy-productdetailpage

Plants

Seed stocks	Wild-type and A17 mutant seeds used in this work were derived from previous studies (references given in Materials and methods section). R108 Tnt1 mutants carrying insertions in MtAnn1 were obtained from Oklahoma State University and are designated ann1-1 (NF0830), ann1-2 (NF17737) and ann1-3 (NF4963). All seeds were propagated locally in adapted plant growth chambers.
Novel plant genotypes	*Describe the methods by which all novel plant genotypes were produced. This includes those generated by transgenic approaches, gene editing, chemical/radiation-based mutagenesis and hybridization. For transgenic lines, describe the transformation method, the number of independent lines analyzed and the generation upon which experiments were performed. For gene-edited lines, describe the editor used, the endogenous sequence targeted for editing, the targeting guide RNA sequence (if applicable) and how the editor was applied.*
Authentication	*Describe any authentication procedures for each seed stock used or novel genotype generated. Describe any experiments used to assess the effect of a mutation and, where applicable, how potential secondary effects (e.g. second site T-DNA insertions, mosiacism, off-target gene editing) were examined.*
